# The Lrp/AsnC-Type Regulator PA2577 Controls the EamA-like Transporter Gene *PA2576* in *Pseudomonas aeruginosa*

**DOI:** 10.3390/ijms222413340

**Published:** 2021-12-12

**Authors:** Magdalena Modrzejewska, Adam Kawalek, Aneta Agnieszka Bartosik

**Affiliations:** Institute of Biochemistry and Biophysics, Polish Academy of Sciences, 02-106 Warsaw, Poland; magda.modrzejewska@ibb.waw.pl (M.M.); a.kawalek@ibb.waw.pl (A.K.)

**Keywords:** *Pseudomonas aeruginosa*, Lrp/AsnC transcriptional regulators, RNA-seq, ChIP-seq, PA2577 regulon, EamA-like transporter

## Abstract

The regulatory network of gene expression in *Pseudomonas aeruginosa*, an opportunistic human pathogen, is very complex. In the PAO1 reference strain, about 10% of genes encode transcriptional regulators, many of which have undefined regulons and unknown functions. The aim of this study is the characterization of PA2577 protein, a representative of the Lrp/AsnC family of transcriptional regulators. This family encompasses proteins involved in the amino acid metabolism, regulation of transport processes or cell morphogenesis. The transcriptome profiling of *P. aeruginosa* cells with mild PA2577 overproduction revealed a decreased expression of the *PA2576* gene oriented divergently to *PA2577* and encoding an EamA-like transporter. A gene expression analysis showed a higher mRNA level of *PA2576* in *P. aeruginosa* Δ*PA2577*, indicating that PA2577 acts as a repressor. Concomitantly, ChIP-seq and EMSA assays confirmed strong interactions of PA2577 with the *PA2577*/*PA2576* intergenic region. Additionally, phenotype microarray analyses indicated an impaired metabolism of Δ*PA2576* and Δ*PA2577* mutants in the presence of polymyxin B, which suggests disturbances of membrane functions in these mutants. We show that PA2576 interacts with two proteins, PA5006 and PA3694, with a predicted role in lipopolysaccharide (LPS) and membrane biogenesis. Overall, our results indicate that PA2577 acts as a repressor of the *PA2576* gene coding for the EamA-like transporter and may play a role in the modulation of the cellular response to stress conditions, including antimicrobial peptides, e.g., polymyxin B.

## 1. Introduction

The Lrp/AsnC family of transcriptional regulators comprises an important group of factors with diverse regulatory functions. The name of the family derives from ‘leucine-responsive regulatory protein’ (Lrp) from *Escherichia coli* and its paralogue—the AsnC protein. Lrp is one of the best-studied bacterial transcriptional regulators which acts as a global factor, considered to control the expression of almost 30% of all *E. coli* genes [1,2]. Lrp action is influenced by leucine and leads to the activation or repression of the transcription of genes affecting a variety of cellular processes, such as amino acid synthesis, the transport of molecules or the formation of pili [2].

The AsnC protein from *E. coli* is a local regulator activating an *asnA* gene that codes for asparagine synthetase A, simultaneously auto-repressing its own gene [3]. The stimulation of *asnA* transcription by AsnC is reduced in the presence of asparagine, being a final product of AsnA action, which represents a classical negative feedback mechanism. Although Lrp and AsnC are probably evolutionarily related (a 25% identity of amino acid sequences), they act divergently, demonstrating an extremely global or specific control [4]. Hence, members of the Lrp/AsnC family of transcriptional regulators may influence cellular metabolism in a global or specific manner, with the second mode of action being dominant among currently characterized Lrp/AsnC-like proteins [5,6,7,8,9].

Lrp/AsnC-type regulators are also known as ‘feast/famine regulatory proteins’ (FFRPs), due to the understanding of their regulatory properties as an adaptation for survival in nutrient-rich or depleted environments [10]. Examples of similarly functioning proteins are widespread in the literature, e.g., PutR from *Agrobacterium tumefaciens* affected by proline, Grp from *Zymomonas mobilis* influenced by glutamate, or MdeR from *Pseudomonas putida* altered by L-methionine [8,11,12].

It has been confirmed by electron microscopy that FFRPs form multimeric assemblies in the presence of genomic DNA [13]. Best characterized representatives of the family form octamers consisting of four dimers [14,15,16,17,18,19,20]. However, other structures of FFRPs have also been described, such as a cylinder formed by six dimers [13]. Both the structures of the regulator and its activation/repression properties can be modulated by a “feast” and “famine” mode, depending on the ligand/effector availability [21,22].

*Pseudomonas aeruginosa* is a pathogen capable of infecting a variety of hosts, including immunocompromised people, and is the object of extensive studies because of its adaptability, allowing survival in different environments. It has previously been reported that *P. aeruginosa* transcriptome is directed by a complex network of direct and indirect links, in which transcriptional regulators comprise a crucial group controlling most cellular processes in response to environmental conditions [23,24]. The high number of genes encoding transcriptional factors belonging to different families (491 genes in the PAO1 reference strain, which represents about 10% of the genome) may provide proof of their importance in *P. aeruginosa* cells. The complexity of gene expression regulation in *P. aeruginosa* can be exemplified by the regulatory network controlling virulence or biofilm formation [25].

There are nine genes encoding Lrp/AsnC-like regulators in the PAO1. Among them, only three have a clarified function. The Lrp protein (PA5308), also known as DadR, is involved in the catabolism of L-alanine and several D-amino acids, regulating the *dadAX* operon. The *dadA* and *dadX* genes code for the D-amino acid dehydrogenase DadA and the amino acid racemase DadX, respectively. DadR is required for the transcriptional activation of *dadAX* and as such induction is enhanced by the presence of intracellular L-alanine [26,27].

The second previously characterized Lrp/AsnC-like regulator from *P. aeruginosa*, KynR (PA2082), directly controls the *kynBU* operon, divergently transcribed to the *kynR* encoding two crucial enzymes of the kynurenine pathway. Kynurenine is a product of tryptophan breakdown, which is converted into anthranilate and intermediates of central metabolism or aromatic compounds. Apart from *P. aeruginosa*, such a pathway of tryptophan catabolism is only present in a small group of bacteria, e.g., *Ralstonia eutropha* or *Bordetella pertussis*, and KynR is highly conserved among them [28].

The transcriptional regulator BkdR (PA2246) is the last member from the family with a known function in *P. aeruginosa* so far. Similarly, as Lrp and KynR, it is also engaged in the control of metabolic processes. BkdR is an activator of the four-gene *bkd* operon divergently transcribed to the *bkdR*. The *bkdA1*, *bkdA2*, *bkdB* and *lpdV* genes encode an enzyme complex involved in the metabolism of valine, leucine and isoleucine by the catalysis of the branched-chain keto acids decarboxylation. The expression of the *bkd* operon is probably stimulated by the presence of these amino acids, which may act as effectors of BkdR [6].

In this work, we report the multilayered characterization of the PA2577 protein from *P. aeruginosa* classified to the Lrp/AsnC family. The *PA2577* gene was previously identified as up-regulated in *P. aeruginosa parA* and *parB* mutants, which are characterized by disturbances in chromosome segregation [23,29,30]. The loci with an altered expression in *par* mutants encompass a significant number of genes encoding transcriptional regulators, often with unknown functions [23]. This study aims to decipher the function of one of them.

*PA2577* is predicted to encode a DNA-binding transcriptional regulator with the molecular size of 16.5 kDa. Its small size as well as domain structure, with the N-terminal helix-turn-helix (HTH) motif involved in interactions with DNA and the C-terminal putative ligand-binding domain, are typical for Lrp/AsnC family members [31].

Our results indicate that PA2577 acts locally as a repressor of *PA2576* gene coding for a predicted membrane protein with two EamA domains. We show that PA2576 interacts with PA3694 and PA5006, which suggests that the function of this protein could be connected to lipopolysaccharide (LPS) biogenesis. The deletion of *PA2577* or *PA2576* led to an increased sensitivity of cells to membrane damaging agents, such as EDTA and polymyxin B. Thus, the proper level of these proteins seems to be crucial for the maintenance of membrane functions important for cell adaptation under stress conditions.

## 2. Results

### 2.1. Genetic Organization of the P. aeruginosa PA2577 Region and Characteristics of PA2577

**Figure 1:**
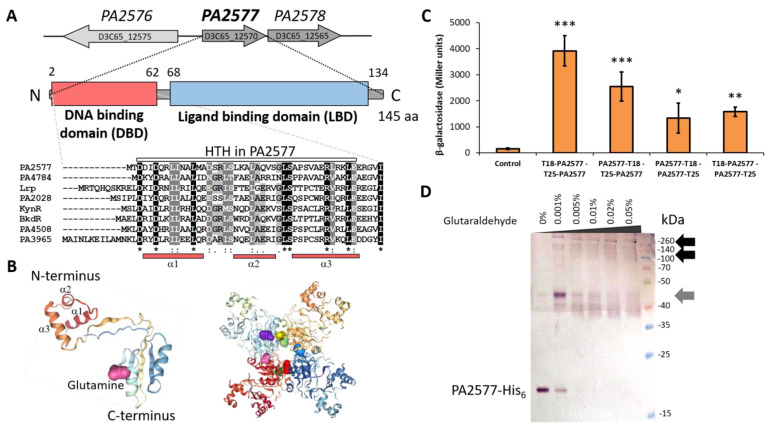
Properties of PA2577 protein from *P. aeruginosa*. (**A**) Genomic context of the *PA2577* gene in the *P. aeruginosa* genome and domain structure of PA2577 protein. The gene names from PAO1 and PAO1161 strains are presented (PAO1 above the arrows presenting loci and PAO1161 inside arrows). Alignment represents a comparison of the PA2577 HTH domain with corresponding regions of 7 Lrp/AsnC proteins from PAO1. Identical residues in all proteins are marked with black. Dots and colons indicate similar residues. Amino acids predicted to be involved in the creation of α-helices are underlined (red bars). (**B**) Predicted structure of PA2577 monomer with marked α-helices creating HTH and an octamer with L-glutamine bound as the ligand. (**C**) BACTH analysis of PA2577 self-interactions. Data represent the mean β-galactosidase activity ± SD in cells from three or more cultures of *E. coli* BTH101 *cyaA* double transformants. Statistical significance was evaluated by *t*-test (* *p*-value < 0.05, ** *p*-value < 0.01, *** *p*-value < 0.001). (**D**) Oligomerization state of purified PA2577-His_6_ assayed by cross-linking with glutaraldehyde. The same amount of purified His_6_-tagged protein was incubated at room temperature for 15 min with indicated glutaraldehyde concentrations. Samples were separated by SDS-PAGE using 12% gel and analyzed by Western blot with mouse anti-His_6_ antibodies. Dimeric forms and oligomeric complexes are marked by grey and black solid arrows, respectively.

The *PA2577* gene of the *P. aeruginosa* PAO1 reference strain is transcribed in an operon with the *PA2578* gene (Figure 1A). *PA2577* and *PA2578*, according to the pseudomonas.com database [32], encode proteins classified as a putative Lrp/AsnC-type transcriptional regulator and a probable acetyltransferase with a GNAT domain, respectively. The divergently transcribed *PA2576* gene encodes a membrane protein with two predicted EamA-like domains. Similar to other members of the Lrp/AsnC family, the PA2577 protein consists of two domains: the N-terminally located DNA-binding domain (DBD) with a helix-turn-helix motif (HTH) and the C-terminal putative ligand-binding domain (LBD) (Figure 1A). The HTH motif is predicted to encompass amino acids 1-63 and is the most conserved part among the members of the family as shown in the DBD sequence comparison between PA2577 and seven other Lrp/AsnC-like regulators from *P. aeruginosa* (Figure 1A). The prediction of the tertiary structure of PA2577 using ColabFold confirmed the two-domain structure of the protein (Figure 1B) [33]. Additionally, the analysis showed high score predictions that L-glutamine could be a possible ligand of PA2577, binding to LBD (Figure 1B). The Swiss-model server [34,35] and HDOCK server [36] were used for the oligo-state assessment and modeling of ligand docking, which resulted in a prediction of PA2577 homo-octamer (Figure 1B). The presented model contained eight molecules of L-glutamine attached to the octamer, one glutamine per one PA2577 monomer. PA2577 LBDs were located in the center of the presented octamer and DBDs outside of it, allowing for DNA binding (Figure 1B).

The bacterial adenylate cyclase two-hybrid (BACTH) system [37] was used to study the dimerization ability of PA2577 in vivo (Figure 1C). The *PA2577* was cloned into two pairs of BACTH vectors, derivatives of pairs pUT18C/pKT25 (pLKB4 and pLKB2) and pUT18/pKNT25 (pKGB4 and pKGB5), facilitating the linkage of tested protein with two *Bordetella pertussis* adenylate cyclase (CyaA) fragments (T18 and T25), either at the N or C-terminus of the protein (Table A1). The reconstruction of adenylate cyclase activity due to the interactions between proteins fused to the CyaA T18/T25 subunits led to the production of cAMP, which bound to the activator CAP and turned on the expression of sugar catabolism genes (e.g., *mal* or *lac* operon). The reconstitution of CyaA activity was evaluated by a change in the color of the colonies on the MacConkey plates (red in the case of interacting proteins; data not shown) and by measurements of β-galactosidase activity in the liquid medium. *E. coli* BTH101 *cyaA* cells transformed with pairs of BACTH vectors (one producing PA2577 fused with subunit T25 and the second with T18) showed very strong PA2577–PA2577 interactions (Figure 1C). The highest activity of β-galactosidase, suggesting the most favorable self-assembly, was detected when the C-ends of proteins were free (T18 and T25 subunits fused with the N-terminal end of PA2577).

Concomitantly, the analysis of the PA2577 oligomerization state using purified PA2577-His_6_ and glutaraldehyde cross-linking confirmed that the purified PA2577 has strong multimerization abilities. Protein dimers as well as higher-order oligomers, possibly octamers, have been observed (Figure 1D). As presented in Figure 1D, results of the cross-linking experiment of PA2577–His_6_ complexes migrated slightly slower than predicted, based on the expected molecular weight (MW) of the protein. The PA2577-His_6_ dimer has molecular weight of about 36 kDa, yet on the membrane, the bands around 40 kDa and 45 kDa were detected. Concomitantly, bands above 140–260 kDa, which may correspond to predicted octamers or even higher-order complexes, were observed. This could be explained by the differential protein solvation by SDS or the hydrophobicity of the protein. The other aspect is the possible presence of post-translational modification(s) or a potential ligand bound to PA2577 and co-purified with the protein upon cross-linking, which could have influenced the MW of PA2577 and formed higher-order complexes. PA2577 seemed to be very potent to multimerization and apart from the dimeric, tetrameric (72 kDa, hardly visible in the lowest glutaraldehyde concentration tested) or octameric form, it could create intermediate forms likely seen as smear or double/multiple bands on the membrane.

Overall, the presented results indicated that PA2577 created higher-order complexes, which is a common property of other Lrp-like proteins [16,18,38].

### 2.2. Effect of PA2577 Lack or Excess on Bacterial Growth

To analyze the role of PA2577 in *P. aeruginosa*, a PAO1161 Δ*PA2577* strain was constructed and used in several phenotypic analyses (e.g., growth rate in M9 and LB medium, biofilm production analysis and motility abilities); however, no significant differences in comparison with the parental strain could be detected (Figure S1 and data not shown).

**Figure 2:**
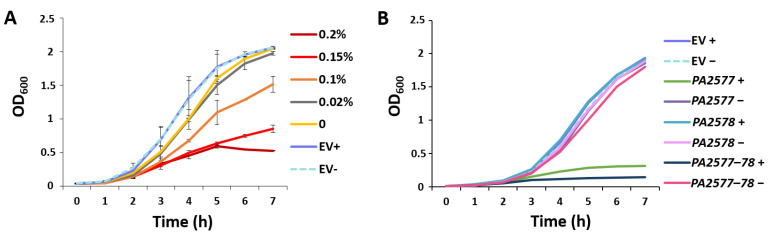
Effect of PA2577 excess on bacterial growth. (**A**) *P. aeruginosa* PAO1161 strains carrying empty vector pKGB8 *araBAD*p (EV) or pMEB12 *araBAD*p-*PA2577* grown in LB under selection with the indicated gradient of inducer concentration (0 to 0.2%). The grey line indicates the growth in the presence of 0.02% arabinose—conditions selected for RNA-seq analysis. Data represent mean OD_600_ from three independent replicates ± SD. (**B**) Comparison of the impact of *PA2577*, *PA2578* and *PA2577*–*PA2578* overexpression on the growth of PAO1161 cells. Strains carrying pAMB9.37 (*lacI^Q^*-*tac*p; EV control), pMEB64 (*lacI^Q^*-*tac*p-*PA2577*), pMEB185 (*lacI^Q^*-*tac*p-*PA2578*) or pMEB186 (*lacI^Q^*-*tac*p-*PA2577*–*78*) were grown in LB under selection with the addition of 0.5 mM (+) or absence (−) of IPTG. Data represent mean OD_600_ from three independent replicates, SD are not shown for clarity.

To check how the overproduction of PA2577 influenced the bacterial growth, strains carrying pKGB8 (*araBAD*p (EV)) or the pMEB12 vector with *PA2577* under the control of the arabinose-induced promoter (*araBAD*p-*PA2577* (PA2577+)) were cultured in a medium containing different concentrations of arabinose. This analysis showed a strong negative effect of PA2577 excess on *P. aeruginosa* growth (Figure 2A). A mild induction of the *PA2577* expression by a very low concentration of arabinose (0.02%) did not have a significant effect on the kinetics of culture growth, whereas using higher concentrations of arabinose resulted in a more pronounced slowdown of bacterial growth (Figure 2A). Concomitantly, a similar analysis of PA2577 overproduction in *E. coli* showed no significant differences between EV and PA2577+ cells upon growth in a medium containing various arabinose concentrations (data not shown), indicating that the effect of PA2577 excess on the slowdown of bacterial growth is specific for *P. aeruginosa*.

Additionally, the effect of the *PA2577*–*PA2578* operon (pMEB186; *lacI^Q^*-*tac*p-*PA2577*–*PA2578* (PA2577-78+)) as well as sole *PA2578* (pMEB185; *lacI^Q^*-*tac*p-*PA2578* (PA2578+)) overexpression was tested. In conditions when the whole *PA2577*–*PA2578* operon was overexpressed, the negative effect on the growth of cells was even stronger than in the case of the sole *PA2577* gene overexpression (Figure 2B). No effect in the case of the *PA2578* protein overproduced alone was detected (Figure 2B).

### 2.3. Transcriptomic Analysis of P. aeruginosa Cells Overexpressing PA2577

**Figure 3:**
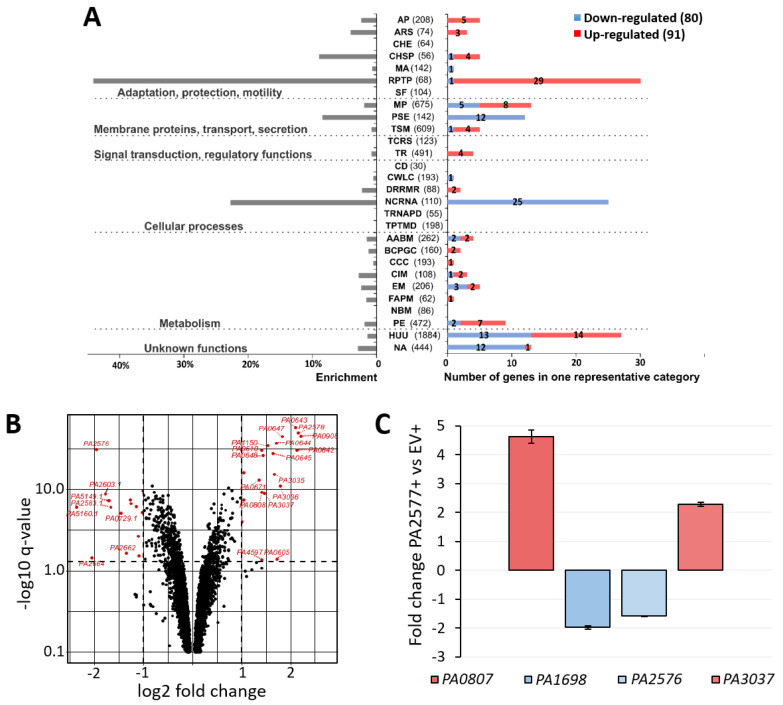
Identification of *P. aeruginosa* genes affected by PA2577. (**A**) Comparative transcriptome analysis was performed for PA2577 overproducing cells (PA2577+) vs. EV+ *P. aeruginosa* PAO1161 cells. Enrichment of PseudoCAP functional categories [32] for 171 genes showing changes in mRNA level in response to mild PA2577 abundance (FC ≤ −1.5 or ≥1.5, FDR adjusted *p*-value ≤ 0.01). The numbers in brackets show the number of all genes in the PAO1 genome in the indicated PseudoCAP category. One gene could be classified into more than one category and one of the most informative categories was selected (bolded in Table S1). Numbers in red or blue bars denote the number of up- or down-regulated genes, respectively, in each category. The PseudoCAP categories were grouped into six classes as described previously [23,39]. AP—adaptation, protection; ARS—antibiotic resistance and susceptibility; CHE—chemotaxis; CHSP—chaperones and heat shock proteins; MA—motility and attachment; RPTP—related to phage, transposon, plasmids; SF—secreted factors; MP—membrane proteins; PSE—protein secretion/export apparatus; TSM—transport of small molecules; TCRS—two-component regulatory systems; TR—transcriptional regulators; CD—cell division; CWLC—cell wall/LPS/capsule; DRRMR—DNA replication, recombination, modification and repair; NCRNA—non-coding RNA genes; TRNAPD—transcription, RNA processing and degradation; TPTMD—translation, post-translational modification, degradation; AABM—amino acid biosynthesis and metabolism; BCPGC—biosynthesis of cofactors, prosthetic groups and carriers; CCC—carbon compound catabolism; CIM—central intermediary metabolism; EM—energy metabolism; FAPM—fatty acid and phospholipid metabolism; NBM—nucleotide biosynthesis and metabolism; PE—putative enzymes; HUU—hypothetical, unclassified, unknown; NA—not annotated. (**B**) Volcano plot visualization of differential expression analysis between transcriptomes of PA2577+ vs. EV+ cells. Each point in the volcano plot represents one gene and the dashed lines represent the cut-off values used. The red dots represent the most significant changes. (**C**) Validation of RNA-seq data by RT-qPCR analysis. The RT-qPCR was performed using RNA samples obtained for the same conditions as samples used for RNA-seq analysis. Data represent mean fold change for three biological replicates vs. mean expression in WT cells.

To identify genes with a changed expression in response to a mild PA2577 overproduction (0.02% arabinose, Figure 2A), an RNA-sequencing (RNA-seq) analysis was performed. Transcriptomes of (PA2577+) cells were compared with those of corresponding cells carrying an empty vector (EV+). Loci specifically altered by a PA2577 excess are listed in Table S1 (fold change (FC) ≤−1.5 or ≥1.5; false discovery rate (FDR) adjusted *p*-value 0.01). The classification of 171 loci with an altered expression was performed according to PseudoCAP functional categories [32], followed by grouping them into six classes as described previously [23,39] (Figure 3A). Additionally, the enrichment of functional categories for differentially expressed genes was performed to point out the most affected functions in response to PA2577. These included phage-related, secretion, chaperones and ncRNA functions. To highlight genes with the highest and the most significant expression alterations, the results of the RNA-seq analysis were visualized as a volcano plot (Figure 3B). Changes in the expression of selected loci (*PA0807*, *PA1698*, *PA2576* and *PA3037*) from the list of genes with an altered expression in response to the PA2577 excess were validated using an independent RT-qPCR analysis (Figure 3C). They correlated with RNA-seq data indicating a decreased (*PA1698* and *PA2576*) or increased (*PA0807* and *PA3037*) mRNA level when comparing PA2577+ with EV+ cells.

Slightly more genes with a significantly changed expression had higher mRNA levels (91 loci). Upregulation was observed for 39 genes coding for predicted bacteriophage proteins (Table S1). The most upregulated gene not related to the cryptic phage was the *PA0807* encoding AmpDh3 protein (Table S1). This protein is one of three AmpD homologs (PA4522, PA5485 and PA0807) in PAO1 repressing the expression of an *ampC* beta-lactamase/cephalosporinase gene [40,41]. Upregulation was also observed for all three genes from the *PA3035*–*PA3037* operon, which is putatively involved in the metabolism of glutathione (Table S1).

Among 80 genes downregulated in response to the PA2577 excess, 25 coded for tRNAs of different amino acids (Ala, Gly, Glu, Thr, Phe, Ser, Arg, Asp, Pro, Leu, Trp, Ile, Tyr and Cys). The *PA5160.1* tRNA gene was the most downregulated locus (FC = 0.2). Among repressed genes, two significantly downregulated loci attracted special attention—*PA2664* and *PA2576* (FC = 0.24 and FC = 0.26, respectively). *PA2664* encodes flavohemoprotein engaged in the oxidation–reduction process. Flavohemoglobins are poorly understood, but flavoHB from *E. coli* is involved in the repair of the lipid membrane caused by oxidative/nitrosative stress [42]. The downregulated *PA2576* gene is also connected with membrane functions. It is oriented divergently to the *PA2577* gene (Figure 1A) and encodes a probable integral inner membrane protein classified as a drug/metabolite transporter based on the existence of two EamA-like domains in its structure. Because of its genomic location in the neighborhood of *PA2577*, the *PA2576* gene was suspected to be the direct target of the PA2577 regulator.

### 2.4. Identification of PA2577 Binding Sites in P. aeruginosa Genome

To check if *PA2576* is indeed the direct target of PA2577 and identify other potential PA2577 binding sites in the *P. aeruginosa* genome, a ChIP-seq analysis was performed. *P. aeruginosa* Δ*PA2577* cells carrying pMEB84 (*lacI^Q^*-*tac*p-*PA2577*-*flag*) were grown under selection in L broth with 0.05 mM IPTG and Δ*PA2577* with pABB28.1 (*lacI^Q^*-*tac*p-*flag*) grown under the same conditions being used as a negative control.

The sequencing of DNA immunoprecipitated using anti-flag antibodies confronted with a negative control revealed as much as 812 binding sites of PA2577. The vast majority of sites was characterized by a very low enrichment and a 2.5-fold enrichment was chosen as a cut-off value, eliminating about 97% of peaks (Figure S2A). Such filtering resulted in 25 peaks considered as specific PA2577 binding sites (Table S2). A total of 23 of these peaks was located between 2 and 5.5 Mb of the genome (Figure S2B). The regions of enrichment had an average length of 52336 bp and the median of 13340 bp, which showed a very low specificity and broad PA2577 interactions with DNA. Due to the broad peaks and different localization of genes with a changed expression (identified in RNA-seq) relative to the peak summits, no specific motif recognized by PA2577 could be identified. In Table S1, RNA-seq data are presented in the first column marked by the number of PA2577 peaks identified in the ChIP-seq analysis if the gene with a changed expression was localized in the region detected as interacting with PA2577. The 81 genes with a changed expression in response to PA2577 were localized in the regions identified as bound by PA2577.

Only four of obtained peaks had sharp shapes with a clear summit and they encompassed regions between *PA0600* and *PA0641* (peak 1), *PA0980* and *PA0975* (peak 14), *PA4334* and *PA4342* (peak 18) and *PA2581* and *PA2573* (peak 25) genes from the PAO1 genome (Table S2, Figure S2C).

Peak no. 1 was 37545 bp long and encompassed the region encoding more than 40 genes (*PA0600–PA0641*), with the summit of the peak identified in the *PA0633* gene encoding a hypothetical protein related to phage functions, such as other genes from the cluster. The induction of the expression of most of the genes from the *PA0612–PA0648* cluster was detected in the RNA-seq analysis in response to a mild PA2577 excess (Table S1). Among these genes were the *prtN* (*PA0610*) and *ptrB* (*PA0612*) encoding transcriptional regulators involved in stress response. Peak no. 14 was 3241 bp long and was the shortest one among 25 peaks identified for PA2577. In this region, the *PA0980–PA0975* genes were located with the *PA0978* encoding conserved hypothetical protein with a putative HTH-like and integrase/transposase catalytic domain, in which the peak summit was detected. However, no changes in gene expression in this region were observed under tested conditions in the performed RNA-seq analysis. Peak no. 18 was 7398 bp long and encompassed the *PA4334–PA4342* genes, with a summit identified in the *PA4336* gene. No changes in gene expression in this region were also observed.

For three PA2577 peaks, the summits were located in intergenic regions (peak 3, 17 and 25) with the highest fold enrichment detected for peak no. 25 encompassing the *PA2576/PA2577* promoter region (Table S2). For peak no. 17, the summit was identified in the intergenic region of the *PA0715/PA0714* genes and a slight down-regulation was observed for the *PA0715* gene encoding a putative RNA-dependent DNA polymerase in response to PA2577 in the RNA-seq analysis (Table S1). Peak no. 3 encompassed the region encoding *PA3061–PA3031* (Table S2). The summit of the peak was detected in the intergenic region of the *PA3035/PA3034* genes. A significant up-regulation of the *PA3035–PA3037* operon was observed in the performed RNA-seq analysis (Table S1). However, an electrophoretic mobility shift assay (EMSA) with the promoter region of *PA3035* showed no clear PA2577 binding to this sequence under tested conditions (data not shown).

**Figure 4:**
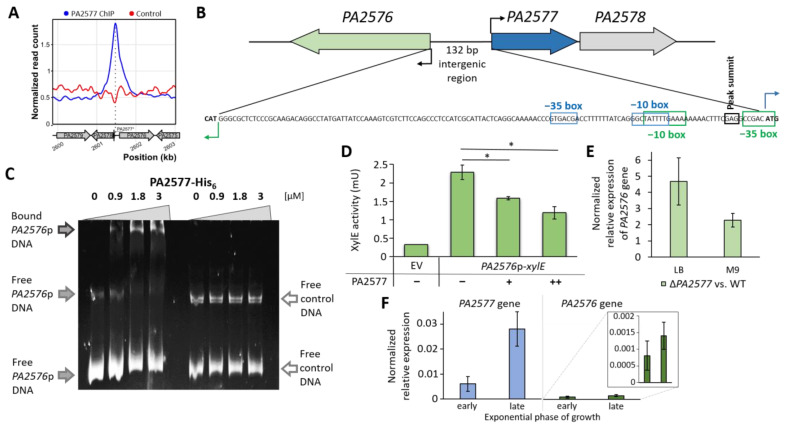
PA2577 interaction with DNA assayed in vivo and in vitro and its regulatory properties. (**A**) ChIP-seq signal over region encompassing PA2577 binding site in *PA2576*/*PA2577* divergent promoter. The plot shows normalized coverage with reads for indicated positions in PAO1161 ∆*PA2577* genome averaged for ChIP replicates. Genes are presented as grey arrows, only names of PAO1 orthologs are shown for clarity. (**B**) Schematic picture of *PA2577*, *PA2576* and *PA2578* loci with the *PA2576*/*PA2577* intergenic region; -35 and -10 regions of *PA2576*p and *PA2577*p predicted using BPROM [43] are marked as well as the summit of ChIP-seq peak. (**C**) EMSA using PA2577-His_6_ and DNA of the *PA2576* promoter region; 100 ng of DNA was incubated with an increasing amount of PA2577-His_6_. Samples were separated on a 10% polyacrylamide gel; 331 bp pCM132 fragment was used as a control to rule out non-specific DNA binding. (**D**) XylE activity in *E. coli* DH5α cells carrying pMEB189 (*PA2576*p-*xylE*) containing pMEB64 (*tac*p-*PA2577*) vector allowing PA2577 overproduction (+) or control pAMB9.37 (−). Strains were grown under selection in L broth. Data for cells with the promoter-less pPTOI (-*xylE*) and pAMB9.37 are shown as a background control (EV). Data represent mean ± SD from three biological replicates. * indicates *p*-value < 0.05 in Student’s two-tailed *t*-test. (**E**) Fold change of *PA2576* expression in Δ*PA2577* vs. WT cells grown in rich (LB) and minimal (M9) medium and harvested at OD_600_~0.5. (**F**) Expression of *PA2577* and *PA2576* genes in PAO1161 in a rich medium at early (OD_600_~0.5) and late exponential phase of growth (OD_600_~1.5). Data represent mean ± SD from three biological replicates.

Peak 25 comprised the *PA2576*/*PA2577* intergenic region (Figure 4A). The summit of this peak was located between the predicted -10 and -35 regions of the *PA2576* promoter close to the predicted *PA2577* transcription start site (Figure 4B). To check the ability of the protein to interact with the *PA2576*/*PA2577* intergenic region, an EMSA using purified PA2577-His_6_ and a DNA fragment encompassing the sequence was conducted. The data indicate that PA2577 recognized and bound to the *PA2576*/*PA2577* intergenic region as it formed a DNA–protein complex, shifting the fragment in the gel (Figure 4C). Such interactions did not occur for PA2577 incubated with the control DNA fragment.

### 2.5. Regulatory Properties of PA2577

To further evaluate the regulatory properties of PA2577, the *PA2577*p and *PA2576*p promoter regions were cloned upstream of a promoter-less *xylE* gene in pPTOI. The *PA2576*p-*xylE* fusion was active in the heterologous host *E. coli* DH5α, whereas no activity was observed for *PA2577*p-*xylE* (Figure 4D and data not shown). The increased expression of *PA2577* in cells carrying plasmids with *PA2576*p-*xylE* resulted in significantly reduced XylE activity in the cell extracts (Figure 4D), indicating that PA2577 acted as a repressor. In accordance with this observation, the RT-qPCR analysis of the *PA2576* transcript level in PA2577-deficient cells showed an increased expression of this gene relative to WT cells grown in rich (LB) or in minimal medium (M9) (Figure 4E).

Moreover, the expression of *PA2577* and *PA2576* was examined at the early (OD_600_~0.6) and late exponential (OD_600_~1.5) phases of growth of *P. aeruginosa* WT cultures. Results of the RT-qPCR analysis revealed that *PA2577* expression was about five times higher in cells collected from the late exponential phase, whereas the *PA2576* gene was expressed at a very low level independently of the phase of growth (about 10 times lower in comparison with *PA2577*) (Figure 4F). Such a low expression level of *PA2576* may suggest that this gene was under strict control involving its local regulator and/or possibly also other proteins. A low increase in the *PA2576* expression was noticed in the late exponential phase even though the *PA2577* was expressed at a higher level. This could be explained by the presence of putative ligand/co-factor, which limited/inhibited the action of PA2577 as the repressor or additional cellular factors, which counteracted the action of PA2577 and were involved in the activation of the *PA2576* expression.

### 2.6. Characterization of PA2577 Target Gene PA2576

The *PA2576* gene encodes the protein predicted to be localized in the inner membrane and possesses two EamA-like domains, each containing five transmembrane helices. To determine the function of the PA2576 protein, a PAO1161 mutant lacking the *PA2576* gene was constructed. Akin to Δ*PA2577* strains, no phenotype changes were observed for the Δ*PA2576* mutant in standard tests (Figure S1). Due to the strong inhibitory effect on *P. aeruginosa* growth caused by the PA2577 overproduction (Figure 2), the influence of *PA2576* overexpression was also tested. The growth analysis of PAO1161 carrying pMEB201 (*lacI^Q^*-*tac*p-*PA2576* (PA2576+)) and pAMB9.37 (*lacI^Q^*-*tac*p (EV)) in medium with IPTG used as the inducer revealed that the overproduction of PA2576 also strongly inhibited *P. aeruginosa* growth (Figure S3).

To further characterize PA2577 and PA2576 proteins, a screening for their cellular partners was conducted using a bacterial two-hybrid system as described previously [44,45]. The genomic library [46] constructed in the pUT18C BACTH vector [44] was used together with CyaA T25-PA2576 (pMEB122) and PA2576-CyaA T25 (pMEB121) or CyaA T25-PA2577 (pMEB67) and PA2577-CyaA T25 (pMEB61) used to produce “bait” proteins. Competent BTH101 cells producing “bait” proteins were transformed with the pUT18C library producing “prey” polypeptides, and possible interactants were identified based on CyaA activity reconstitution [45]. Despite the extensive screening of the library, no interactants of PA2577 could be identified with this method.

**Figure 5:**
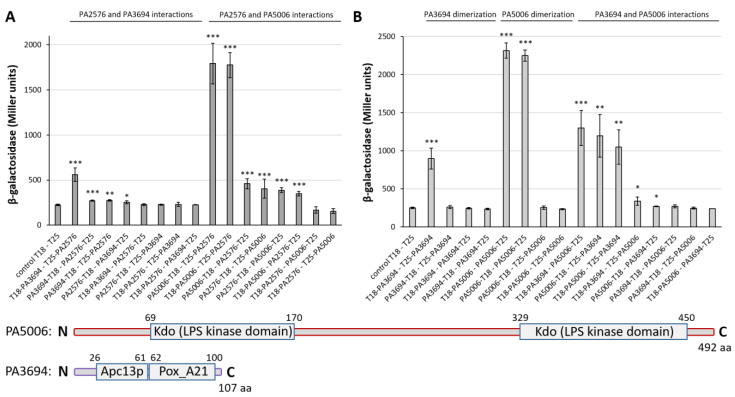
BACTH analysis of (**A**) PA2576 interactions with its two identified partners—PA5006 and PA3694—and (**B**) PA5006 and PA3694 self-interactions and interactions between each other. Data represent the mean ± SD values of β-galactosidase activities from three independent cultures of *E. coli* BTH101 *cyaA*^−^ double transformants. Statistical significance was evaluated by *t*-test using measurements for transformants carrying double empty vectors as a control (* *p*-value < 0.05, ** *p*-value < 0.01, *** *p*-value < 0.001). The schematic domain structure of PA5006 and PA3694 proteins (based on the Kyoto Encyclopedia of Genes and Genomes (KEGG) database) is presented below the charts.

Concomitantly, the screening for T25-PA2576 interactants revealed two proteins, PA3694 and PA5006. A full-length PA3694 protein was encoded by three independent clones, whereas a 579 bp long part of *PA5006* (encoding fragment encompassing 300-493 aa) was identified in one clone. The subsequent cloning of a full-length *PA3694* and *PA5006* into the BACTH vectors confirmed strong interactions between PA5006 and PA2576 and medium-strength interactions between PA3694 and PA2576 (Figure 5A). The strongest values of β-galactosidase activity were observed for variants of CyaA T25 fused with the N-terminal end of PA2576 (Figure 5A), which confirmed that the interaction with the C-terminal part of PA2576 was the most preferable way of the interplay. The significance of all interactions was confirmed by control tests of β-galactosidase activity performed with an empty BACTH vector and appropriate protein fused with a *cyaA* subunit, where no interactions were observed (Figure S4).

### 2.7. Characteristics of PA2577 Network and Its Role in P. aeruginosa

To better characterize the identified partners of PA2576, the ability of PA3694 and PA5006 to dimerize and to interact with each other was tested. Results of a BACTH assay followed by β-galactosidase activity testing revealed that both PA3694 and PA5006 were able to dimerize regardless of which fusion N- or C-terminus of the protein fused with the T18 or T25 CyaA subunit was tested (Figure 5B). What was even more interesting, partners of PA2576 also interacted with themselves (Figure 5B), which suggested the presence of a protein complex consisting of at least three members (PA2576/PA3694/PA5006). For the PA2576 protein, no dimerization was observed (Figure S5).

PA3694 is an uncharacterized small protein probably being a part of an operon of unknown function consisting of six genes (*PA3693–PA3698*). Based on the amino acid sequence, it is predicted to be a lipoprotein [32].

*PA5006* is the first gene from an operon consisting of seven loci (*PA5006*–*PA5012*) [47]. *PA5006* is the only putative gene in the cluster and probably the same as three other genes (*waaP* (*PA5009*), *wapP* (*PA5008*) and *wapQ* (*PA5007*)) belonging to the operon, encoding LPS kinase, which is responsible for the addition of phosphates to the inner core oligosaccharides of *P. aeruginosa* LPS (Figure 5). The four kinase genes occur in the cluster together with LPS sugar transferase genes *waaF*, *waaC* and *wapG* (*PA5010*–*PA5012*) creating the operon encoding proteins responsible for the construction of the *P. aeruginosa* LPS core, which is built of highly phosphorylated sugars [48,49]. LPS phosphates are necessary for a complete LPS synthesis before its transport to the outer membrane of the cell [47].

**Figure 6:**
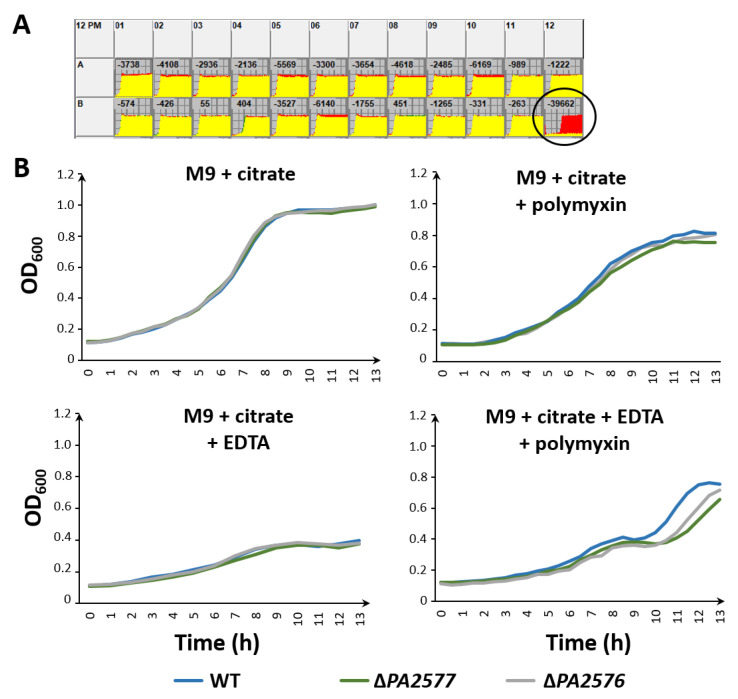
(**A**) BIOLOG phenotype microarray results of Δ*PA2576* strain vs. WT strain on plate PM12B. B12 location corresponding to conditions with the highest concentration of polymyxin B is marked. (**B**) Growth curves of the *P. aeruginosa* PAO1161 Δ*PA2577*, Δ*PA2576* mutants and WT strain in M9 supplemented with citrate, with the addition of polymyxin B (1 µg/mL) or/and EDTA (0.5 mM) at 37 °C. Data represent mean OD_600_ from three independent replicates.

In light of the BACTH results and the existence of an interaction network comprising PA2576/PA3694/PA5006, phenotypic microarrays (BIOLOG) for selected conditions for the Δ*PA2576* and Δ*PA2577* mutants in comparison with the WT strain were performed (plates PM3, PM4, PM5, PM9 and PM13), but no significant differences could be observed in such conditions (data not shown). A phenotype microarrays analysis conducted on plate PM12B indicated an impaired metabolism of the Δ*PA2576* mutant in the presence of polymyxin B under the highest tested concentrations, which suggested disturbances of membrane functions likely connected with LPS synthesis (Figure 6A).

The phenotype was confirmed by a standard growth experiment where PA2576- or PA2577-deficient cells grew significantly slower than the WT strain in the minimal M9 medium supplemented with citrate as the carbon source, polymyxin B and EDTA (Figure 6B).

Overall, presented data indicated that PA2577 and PA2576 were involved in the control of membrane functions, which might be perturbed in the presence of agents influencing membrane integrity, e.g., polymyxin B.

## 3. Discussion

In this work, the characterization of the Lrp/AsnC transcriptional regulator PA2577 from *P. aeruginosa* was described. Representatives of the Lrp/AsnC family of regulators appeared to be widely distributed among bacteria and archaea, whereas no Lrp-like TRs were identified in eukaryotic genomes so far [31]. Some prokaryotes encoded only a few (or even none) representatives of the Lrp/AsnC TRs family, e.g., *E. coli* with three these types of regulators (Lrp, AsnC and YbaO), but several bacteria contained dozens of such paralogues [31]. Different compositions of exact families of TRs may be related to the lifestyle, metabolic capabilities and adaptation/survival ability of the bacterial species. The higher number of regulators encoded in the genome often reflects the need to control and coordinate various cellular processes; thus, the complexity of the regulatory and metabolic network allowing adaptation and survival in a changing environment. In the genome of *P. aeruginosa*, known for its high adaptability, nine Lrp/AsnC-type regulators could be identified (PA0515, PA2028, PA2082, PA2246, PA2577, PA3965, PA4508, PA4784 and PA5308).

Our work showed that PA2577 could form multimers, bind DNA and modulate gene expression in *P. aeruginosa*. An in silico analysis predicted a two-domain structure of PA2577, typical for FFRP, with the N-terminus involved in DNA binding and C-terminal ligand binding domain [31]. The possibility to create octamers was envisioned by the modeling of a PA2577 3D structure based on available 3D structures (Figure 1B). In the model, four dimers created an octamer with C-terminal parts of the PA2577 dimers located in the center and DBDs positioned outside of the multimer; thus, allowing interactions with DNA. In the presented model, each PA2577 monomer bound one glutamine; however, the binding of this ligand to PA2577 has not yet been tested experimentally. Studies on the representatives of Lrp/AsnC family members showed that ligand binding may not be necessary to create multimers, such as in Grp from archaeon *Sulfolobus tokodaii* [50] or DM1 from *Pyrococcus* sp. OT3 [51]. In fact, ligands were shown to either stabilize the multimer structures as in the case of the DM1 protein or Grp [10,50] or destabilize its integrity similar to how leucine acts on Lrp protein [52].

PA2577 showed a 27% identity and 49% similarity with Grp, and conserved amino acids involved in interactions with glutamine in Grp seemed to also be present in PA2577, e.g., S31, Y62, D100, T127 and T129 [50]. In the case of Grp, octamers were formed independent of the ligand, but conformational changes after glutamine binding might have caused the stabilization of the oligomeric structure and modulated protein-DNA interactions [50]. Especially important seemed to be the residues T132 and T134 (T127 and T129 in PA2577), which were highly conserved in most Lrp/AsnC family members. They possibly played a role in the dimer–dimer interface stabilization in the presence of the ligand, which would further reinforce the quaternary structure and possibly exert an effect on the regulatory properties of the protein.

The formation of oligomeric structures by Lrp/AsnC proteins played a pivotal role in their interactions with DNA, sometimes allowing drastic conformation changes of the DNA duplex, such as DNA bending/wrapping [50,53,54,55], as described for the Lrp dimer [55,56]. DNA bending at a close proximity of the -10 promoter sequence might be involved in transcription control, especially activation [57,58,59].

The performed RNA-seq analysis showed changes in the expression of 171 genes in response to PA2577. We did not observe a clear correlation of identified PA2577 binding sites in ChIP-seq (using a 2.5-fold enrichment cut-off value) and genes that responded to PA2577 in the RNA-seq analysis under tested conditions, except for *PA2576*. Under tested conditions, PA2577 acted as the repressor of the *PA2576* gene, transcribed divergently to *PA2577*.

The intergenic region of *PA2576/PA2577* genes consisted of tracts of T and A bases, which may have helped in the modulation of the promoter structure by the bound PA2577 oligomer, possibly octamer, according to the model structure, and possible DNA bending and wrapping similar to LrpC from *B. subtilis* [53]. The AAAGTTTTTTTTCAAAATA tract encompassed a -10 box of predicted *PA2576*p as well as *PA2577*p (Figure 4B). Additionally, A and T tracts between the -10 box and the start codon of *PA2576* were present in the *PA2576/PA2577* intergenic region, which may also have assisted in the promoter structure modulation after PA2577 binding to exert an effect on gene regulation. Additionally, the pseudopalindromic sequence CCTGATAAAAAAGG was identified in this region, which may have helped in specific recognition by PA2577. The palindromic sequences were preferentially recognized by dimeric forms of regulatory proteins, allowing specific interactions between DNA and protein [60], e.g., the dimer binding consensus sequence for the FL11 from *Pyrococcus* OT3 was ATAAA ATT TTTAT. Additionally, for many Lrp/AsnC homologs, a few similar homologous sequences with some periodicity (7–8 bp or 18 bp or 30–31 bp) are usually present in the target gene promoters [13]. This may explain the interactions of octamers by facing out DNA binding domains with such arranged DNA sequences and the possibility to wrap DNA around a protein oligomer and the creation of higher-order nucleoprotein complexes.

Based on transcriptomic profiling, PA2577 might be indirectly involved in the control of several genes, e.g., *ampDh3* or the *PA3035–PA3037* operon. Intriguing was the observed decrease in the tRNA gene expression or upregulation of genes related to phages in response to PA2577 overproduction (Table S1). The mechanism of these changes remains unclear, but we assumed that it could be the cellular response to the stress effect of *PA2577* overexpression on bacterial growth (the inhibition of protein synthesis processes), even though the concentration of inducer was low. The induction of the expression of the *PA0612*–*PA0648* gene cluster is often observed under different stress-generating growth conditions [23,39,61]. Alterations of tRNA gene expression may also account for indirect effects of the PA2577 excess, which was suggested by the results of the ChIP-seq analysis where no binding of PA2577 to the promoters of these loci could be observed.

Genes upregulated in response to PA2577 encompassed the *ptrB* encoding protein known as a repressor of the type III secretion system (T3SS) under the stress of DNA damage; hence, the observed effect of the down-regulation of T3SS genes might be an indirect effect of PtrB action [62]. Similarly, the downregulation of the *exsE* may exert an effect on T3SS genes, as ExsE acts as an anti-anti-activator, which interacts with ExsC, a positive regulator of the type III secretion regulon [63]. Similarly, the upregulation of the *PA3035*–*PA3037* operon, where *PA3035* encodes glutathione S-transferase, may be connected with transport functions, since such transferases are engaged in detoxification processes [64].

Our in vitro and in vivo studies showed that PA2577 was directly involved in the regulation of the divergent gene *PA2576* encoding a drug–metabolite transporter (DMT) from the EamA family PA2576. The action of PA2577 resembled other local regulators from the Lrp/AsnC family being divergently transcribed in respect to the target gene, e.g., AsnC from *E. coli* [3,65], BkdR and MdeR from *P. putida* [6,8], PutR in *Agrobacterium tumefaciens* [11] or PutR in *Rhodobacter capsulatus* [5].

The DMT superfamily of proteins comprises 26 families [66]. The EamA family is one of the members of the DMT-like transporters and is named after the O-acetylserine/cysteine export gene from *E. coli*. In the structure of the representatives of the EamA family, two identical parts comprising individual EamA domains could be distinguished. It is most probably an effect of the intra-protein domain duplication event during evolution [67]. The function of only a few EamA-like representatives is described in the literature, e.g., YdeD from *E. coli* being an archetype for the family, but also PecM from *Erwinia chrysanthemi* [68,69]. Many family members are involved or predicted to be engaged in the amino acid metabolism, but there is also a branch of nucleotide sugar transporters (NSTs), e.g., the solute carrier family SLC35 [70].

Although we were not able to identify the cargo of the PA2576 transporter, our studies demonstrated its interactions with the putative lipoprotein PA3694 and putative kinase PA5006. These interactions may partially explain the observed disturbances in the growth of the *P. aeruginosa* Δ*PA2576* mutant in the presence of polymyxin B and EDTA. This was connected with the increased sensitivity of the *PA2576* mutant to agents acting on bacterial membranes. This linked the action of PA2576 with the proper maintenance of membrane functions, homeostasis and LPS biosynthesis through PA5006.

**Figure 7:**
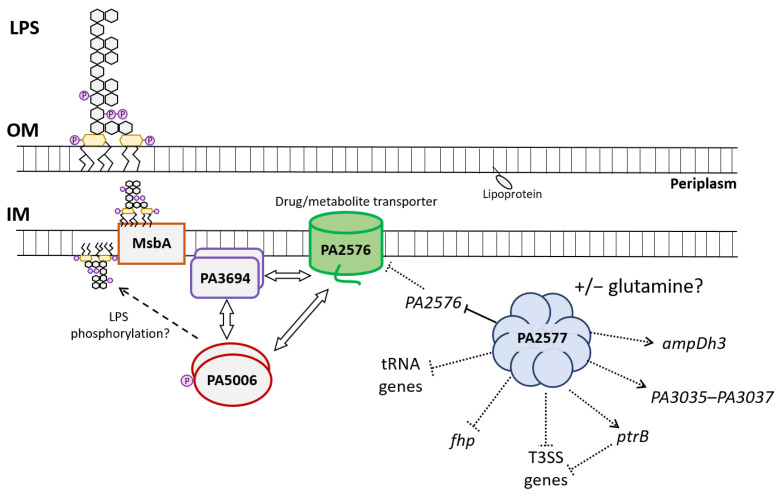
Schematic picture presenting relationships between PA2577 regulator, PA2576 transporter and partners of PA2576 probably existing in dimeric forms in the cytoplasm. PA2577 regulator is presented as an octamer. IM—inner membrane; OM—outer membrane; LPS—lipopolysaccharide. A solid line indicates direct repression by PA2577; dotted lines indicate direct and/or indirect involvement of PA2577 in gene expression control.

Based on the presented results, the model of the regulatory network engaging PA2577, PA2576 and the partners in *P. aeruginosa* was proposed (Figure 7). PA2577 could create multimers and act as the local regulator of the *PA2576* gene. It repressed the expression of *PA2576*, which possibly diminished the PA2576 pool in the cell and led to disorders in transport function and interactions with partner proteins PA3694 and PA5006. PA2576–PA5006–PA3694 created a protein complex, possibly next to the inner membrane, and these interactions may influence putative PA5006 kinase/phosphotransferase activity involved in LPS inner core oligosaccharides phosphorylation. LPS inner core phosphates are necessary for a complete LPS biogenesis, its transport to the periplasm via MsbA (PA4997) and further to the outer membrane [47]. MsbA was shown to translocate components of mature LPS to the periplasmic side of the membrane [71]. The MsbA protein is an ABC transporter that is crucial for the transport of the lipid A-core, being synthesized at the cytoplasm close to the inner membrane [71]. A lipid A-core is most probably transported in a phosphorylated form from cytoplasm, where LPS kinases mediate the addition of phosphate groups [47]. PA2576 as an interactant of phosphate kinase PA5006 possibly participates in this poorly understood process of LPS phosphorylation during biosynthesis. LPS plays an important role in the maintenance of the outer membrane integrity and is considered as a virulence factor and one of the strongest antigens inducing host immune response during bacterial infection [71].

As indicated transcriptomic profiling PA2577 might be indirectly involved in the negative regulation of genes encoding a type III secretion system, flavohemoprotein, tRNAs as well as in the activation of the *ampDh3* and the *PA3035*–*PA3037* genes (Figure 7). The T3SSs are widely distributed in Gram-negative bacteria and play a pivotal role in pathogen–host interactions during plant, animal or human infections caused by pathogenic bacteria [72]. The T3SS is a key virulence factor composed of a surface-attached needle-like complex that is able to inject cytotoxins directly into host cells, causing cellular damage and, ultimately, death. In *P. aeruginosa*, the T3SS is expressed by planktonic bacteria during acute infection. Its expression is tightly controlled and induced in response to several environmental signals, including contact with the host cell or low extracellular calcium concentration [73]. Interestingly, it is suggested that structural changes in the LPS of *P. aeruginosa* would affect the ability of bacteria to efficiently secrete cytotoxins and elicit an acute infection. This would be connected with changes in the expression of components of the T3SS; specifically, a less structured LPS lacking the LPS A-band or B-band O antigen would promote cytotoxin production and secretion by bacterium [74]. PA2577 with a possible role in the modulation of LPS synthesis and T3SS expression might be involved in this regulatory cascade. The action of PA2577 could be modulated by the ligand availability, as in the case of other Lrp/AsnC family members [31]. Further investigations have to be performed to unravel the exact mechanism of the PA2577/PA2576/PA5006/PA3694 action. The proposed model (Figure 7) of relationships between PA2577 and its targets points out the directions of future studies and explorations.

## 4. Materials and Methods

### 4.1. Growth Conditions and Bacterial Strains and Plasmids

Bacterial strains used and constructed in this study are listed in Table A1. Strains were grown in LB or on LB agar at 37 °C. *P. aeruginosa* strains were also cultivated in M9 minimal medium supplemented with sodium citrate (0.25%) as the carbon source and leucine (10 mM) for *leu*^−^ strains. For plasmid selection in *E. coli*, media were supplemented with 10 µg/mL chloramphenicol, 50 µg/mL kanamycin or benzyl penicillin at a final concentration of 150 µg/mL in liquid medium or 300 µg/mL in agar plates. For *P. aeruginosa* strains, the following antibiotics and concentrations were applied: carbenicillin (300 µg/mL), rifampicin (300 µg/mL), kanamycin (250 µg/mL in a liquid medium; 500 µg/mL in plates) and chloramphenicol (75 µg/mL in a liquid medium; 150 µg/mL in plates). For phenotype growth tests, M9 medium supplemented with EDTA in a concentration of 0.5 mM and polymyxin B in a concentration of 1 µg/mL was used.

Competent *E*. *coli* cells were prepared with the use of the CaCl_2_ method and transformation was conducted according to a standard procedure [75]. Competent *P*. *aeruginosa* cells were prepared as described earlier [76].

All plasmids used and constructed in this study are described in Table A1.

Overexpression of selected genes was examined by the transformation of *P. aeruginosa* PAO1161 WT or mutant strain with the pAMB9.37, pABB28.1 or pKGB8 derivatives.

*P. aeruginosa* PAO1161 Δ*PA2577* and Δ*PA2576* chromosomal mutants were obtained by the method of allele exchange [29]. The donor strain was obtained by transformation of competent *E. coli* S17-1 cells with pMEB17 or pMEB164 plasmid (derivatives of suicide vector pAKE600). *P. aeruginosa* PAO1161 Rif^R^ was used as the recipient. After conjugation, selection and screening of transconjugants were performed by the growth on LB plates with rifampicin and carbenicillin. Removal of the vector was conducted by overnight growth in LB with 10% sucrose. Selection of colonies with exchanged allele was performed by PCR using primer pairs #3/#6 and #10/#13 (Table A2).

### 4.2. Growth Experiments

For tests of growth kinetics, liquid media were inoculated with strains propagated on plates. Overnight cultures were diluted 1:100 in a fresh medium and then incubated at 37 °C whilst shaken. When the experiment was performed in M9 medium, overnight cells grown in LB were washed twice with M9 medium and then used for inoculation. Bacterial growth was monitored by the measurement of optical density at 600 nm (OD_600_) in a spectrophotometer or with the use of Varioskan Lux Multimode Microplate Reader and SkanIt RE software (Thermo Fisher Scientific, Waltham, MA, USA) in the case of growth in 96-well plates.

### 4.3. Analysis of Protein–Protein Interactions in the Bacterial Two-Hybrid System

The bacterial adenylate cyclase two-hybrid (BACTH) system was used to analyze protein–protein interactions in vivo [37,44]. CyaA-T18 and CyaA-T25 fragments were fused to the indicated termini of tested proteins in vectors: pLKB2 and pLKB4 or pKGB4 and pKGB5 (Table A1). Constructs in pLKB plasmids were prepared to obtain N-terminal fusions of cloned genes with *cyaA* subunits, whereas pKGB derivatives encoded genes without stop codon fused with *cyaA*-T18 and *cyaA*-T25 in the C-terminus. Details of cloning design are described in Table A1. Pairs of complementary plasmids were used to co-transform *E. coli* BTH101 *cyaA*^−^ cells. Double transformants were plated onto MacConkey agar supplemented with 1% maltose, 0.1 mM IPTG, kanamycin and penicillin, and grown at 30 °C for 48 h. Randomly picked colonies for each variant of transformation were streaked on a fresh MacConkey plate and again incubated at 30 °C for 48 h. Clones were used to inoculate liquid cultures in L broth and after overnight growth in 37 °C, and the β-galactosidase activity assay was performed in cell extracts as previously described [77]. BACTH system was used in the tests of protein in vivo self/association and for the identification of interactions between two proteins.

### 4.4. Motility and Biofilm Formation Assays

Motility assays were performed as described previously [78]. To standardize the assays, all plates were prepared with the same volume of the medium. Biofilm amount was measured with the crystal violet staining method in a rich medium [79].

### 4.5. RNA Isolation, RNA-Seq and RT-qPCR

Strains obtained by transformation of PAO1161 with pMEB12 or pKGB8 were used for total RNA preparation. RNA isolation and sequencing as well as data analysis were performed essentially as previously described [79]. Raw data are available in the NCBI’s Gene Expression Omnibus (GEO) database (http://www.ncbi.nlm.nih.gov/geo/ (accessed on 30 October 2021)) under accession number GSE186749.

Expression changes for selected genes were confirmed by RT-qPCR using RNA isolated from independent cultures. In total, 1 µg of each RNA sample was used for reverse transcription (TranScriba Kit, A&A Biotechnology, Gdańsk, Poland) with the use of random hexamers. Three technical replicates of PA2577 overproducer and EV control were used. The *nadB* was used as the reference. RT-qPCR was also used for examination of the expression of *PA2577* and *PA2576* in the early (OD_600_~0.5) and late exponential (OD_600_~1.5) phases of growth of *P. aeruginosa* WT cultures and for *PA2576* also in *P. aeruginosa* Δ*PA2577* strain. The *rpsL* gene was used as the reference. Relative gene expression was calculated using the Pfaffl method [80]. All oligonucleotides used in RT-qPCR are listed in Table A2.

### 4.6. Chromatin Immunoprecipitation with Sequencing

ChIP was performed as previously [81,82]. *P. aeruginosa* Δ*PA2577* strain carrying pMEB12 (*araBAD*p-*PA2577*-*flag*) or pKGB8 vectors was grown in a medium containing 0.02% arabinose as the inducer. Additionally, it was used in the ChIP procedure with anti-flag antibodies (MA1-91878, Invitrogen). Data were processed essentially as previously [82]. Sequencing data are available in the NCBI’s Gene Expression Omnibus (GEO) database (http://www.ncbi.nlm.nih.gov/geo/ (accessed on 30 October 2021)), under accession number GSE186746.

### 4.7. Protein Overproduction and Purification

To purify His_6_-tagged PA2577 protein, *E. coli* BL21(DE3) strain was transformed with pMEB105. Cells were grown overnight in LB broth at 37 °C with 0.5 mM IPTG and then harvested by centrifugation. Phosphate buffer (50 mM sodium phosphate with 300 mM NaCl, pH 8.0) supplemented with lysozyme at a final concentration of 1 mg/mL and 1 mM PMSF was used for resuspension of the pellet. The supernatant obtained after sonication and centrifugation was transferred onto Ni-agarose columns (Protino Ni-TED 1000, Macherey-Nagel) and purification of the PA2577-His_6_ was carried out according to the manufacturer’s instructions. The gradient of imidazole (20, 100, 250 mM) was used for elution. Purified proteins were dialyzed by overnight incubation in phosphate buffer containing 10% (*v*/*v*) glycerol and stored in small aliquots at −80 °C. Purification was monitored by SDS-PAGE with a Pharmacia PHAST gel system.

### 4.8. Cross-Linking with Glutaraldehyde

Purified PA2577-His_6_ was cross-linked with increasing concentrations of glutaraldehyde as previously described [83]. Samples were then suspended in the loading buffer (50 mM Tris-HCl (pH = 8.0), 0.1 M DTT, 2% SDS, 0.1% bromophenol blue, 10% glycerol), boiled for 5 min and separated on 12% (*w*/*v*) SDS-PAGE gels. After overnight wet transfer onto nitrocellulose membranes (Amersham Protran, Cytiva, Marlborough, MA, USA), Western blot analysis was performed with anti-His_6_ mouse antibodies.

### 4.9. In Vitro Protein–DNA Interactions

Electrophoretic mobility shift assay with purified PCR products and PA2577-His_6_ was conducted in binding buffer (10 mM Tris-HCl (pH = 8.5), 10 mM MgCl_2_, 100 mM KCl, 0.1 mg/mL BSA). DNA was amplified with the use of #16/#21 primers for *PA2576* promoter and #20/#21 primers for non-specific DNA. pMEB78 and empty pCM132 were used as templates. DNA was stained by ethidium bromide and visualized by UV light. *PA2576*p region encompassed the whole 132 bp intergenic fragment between *PA2576* and *PA2577*. The complexes were separated on a 10% polyacrylamide gel in 0.5 × Tris-borate-EDTA (TBE) buffer.

### 4.10. Regulatory Experiments and Promoter Activity Tests

*E*. *coli* DH5α carrying pPTOI derivatives with a promoter fused to the *xylE* reporter gene together with pMEB64 (*lacI^Q^*-*tac*p-*PA2577*) or pAMB9.37 (*lacI^Q^*-*tac*p) were assayed for catechol 2,3-oxygenase activity (the product of *xylE* gene). The experiment was performed as previously described [84] using cell extracts prepared from exponentially growing cultures in L broth. Measurements of the amount of the protein in extracts were indicated by the Bradford assay [85].

### 4.11. Screening for Protein Partners

For the search and screening of protein interactants, the available PAO1161 genome library constructed in the pUT18C vector (carrying T18 *cyaA* fragment) was used [46]. Screening of partners was performed based on the previously described procedure using protein–protein interactions in the bacterial two-hybrid system [37,45]. *PA2577* and *PA2576* genes were cloned in the pLKB2 and pKGB5 plasmids to obtain proteins fused with the T25 *cyaA* subunit as described in Table A1. Competent BTH101 cells carrying pMEB121 or pMEB122 and pMEB61 or pMEB67 were prepared using the CaCl_2_ method and used for transformation with pUT18C, containing random fragments of *P. aeruginosa* genomic DNA. The mixture obtained after the transformation procedure was washed with sterile water to discard the rich medium and, after that, plated onto M9 minimal medium supplemented with kanamycin, penicillin, 75 µM thiamine, 1% maltose, 40 µM X-gal and 0.1 mM IPTG. Plates were incubated for 5 days at 30 °C and all blue colonies indicating the interaction between T18 and T25 *cyaA* subunits and reconstitution of CyaA activity were re-streaked on the same medium. Plasmid DNA isolated from colonies that remained blue after re-streaking was used to perform the transformation of *E. coli* BTH101 cells carrying empty pLKB2 or pMEB122/pMEB67. The red color of colonies obtained on MacConkey plates for the transformation of cells carrying PA2576 or PA2577 and white colonies in the case of cells with an empty vector were presumed as the confirmation of identified interactant. Plasmid DNA containing a fragment of positively verified putative partners was sequenced. Genes encoding identified partners were then cloned into the BACTH vectors and interactions with full-length proteins were tested.

### 4.12. Phenotype Microarrays Analysis

Phenotypic Microarray Plates (PM3, PM4, PM5, PM9, PM12B and PM13) (Biolog Inc., Hayward, CA, USA) were used to test the growth capacity in different conditions of the analyzed PAO1161 WT, Δ*PA2576* and/or Δ*PA2577* strains as previously described [86,87]. *P. aeruginosa* strains taken from −80 °C stocks were grown on L agar plates at 37 °C and used to prepare inoculation cultures. The analysis was repeated twice for each plate.

## Data Availability

Raw data of RNA-seq and ChIP-seq are available in the NCBI’s Gene Expression Omnibus (GEO) database under accession numbers GSE186749 and GSE186746.

## Supplementary Materials

The following are available online at https://www.mdpi.com/article/10.3390/ijms222413340/s1.

## Appendix A

**Table A1:** Bacterial strains and plasmids used and constructed in this study.

**Strain Name**	**Description**	**Reference**
* Escherichia coli *		
DH5α	F− Φ80*lacZ*Δ*M15* Δ(*lacZYA-argF*) *U169 recA1 endA1 hsdR17* (r_K_−, m_K_^+^) *phoA supE44 λ thi-1 gyrA96 relA1*	[88]
S17-1	*pro* Δ*hsdR hsdM*^+^ *recA* Tp^R^ Sm^R^ ΩRP4-Tc::Mu Kn::Tn7	[89]
BL21	F− *ompT hsdS*_B_ (r_B_^−^m_B_^−^) *gal dcm* (λ DE3)	Novagen
BTH101	F− *cya-99 araD139 galE15 galK16 rpsL1* (Str^R^) *hsdR2 mcrA1 mcrB1*	[37]
*** Pseudomonas aeruginosa ***		
PAO1161	PAO1161 Rif^R^ *leu*−, r−, m−	[30]
PAO1161	PAO1161 Rif^R^ r−, m−	[90]
PAO1161 Δ*PA2577*	PAO1161 Rif^R^ with deleted gene *PA2577*, allele exchange with the use of pMEB17	This study
PAO1161 Δ*PA2576*	PAO1161 Rif^R^ with deleted gene *PA2576*, allele exchange with the use of pMEB164	This study
**Plasmid Name**	**Description**	**Reference**
pBBR1-MCS-1	Cm^R^, IncA/C broad-host-range cloning vector, *lacZ*α–MCS, *mob*, T7p, T3p	[91]
pAMB9.37	pBBR1-MCS-1 derivative with lacI^Q^-*tac*p, expression vector	[92]
pABB28.1	pBBR1-MCS-1 derivative with *lacI^Q^*-*tac*p-*flag*, expression vector	[82]
pAKE600	Ap^R^, *ori*_MB1_, *oriT*_RK2_, *sacB*, suicide vector	[93]
pBGS18	Km^R^, *ori*_MB1_, cloning vector	[94]
pCM132	Km^R^, *oriC*_ColE1_, *oriV*_IncP_, *oriT*, *traJ*’, *trfA*, broad-host-range vector, promoter-less *lacZ* reporter gene	[95]
pET28a(+)	Km^R^, *ori*_MB1_, T7p, *lacO*, His_6_-tag, T7 tag, expression vector	Novagen
pPTOI	Km^R^, *oriV*_pSC101_, promoter-less *xylE* cassette	[96]
pET28mod	Km^R^, *ori*_MB1_, T7p, *lacO*, His_6_-tag, modified to remove T7 tag	[97]
pKAB240	Ap^R^, *ori*_MB1_, pUC19 derivative with His_6_-*mcs* (MunI, HindIII, NotI, XhoI, BamHI)-*flag*	[82]
pKGB8	Cm^R^, broad-host-range expression vector, ori_IncA/C_, *araC*-*araBAD*p	[39]
pUT18C	Ap^R^; *ori*_ColE1_, *lac*p–*cyaT18*–*mcs*	[37]
pUT18	Ap^R^; *ori*_ColE1_, *lac*p–*mcs*–*cyaT18*	[37]
pKT25	Km^R^, *ori*_p15_, *lac*p–*cyaT25*–*mcs*	[37]
pKNT25	Km^R^; *ori*_p15_, *lac*p–*mcs*–*cyaT25*	[37]
pLKB4	pUT18C with modified *mcs*	[98]
pKGB4	pUT18 with modified *mcs*	[98]
pLKB2	pKT25 with modified *mcs*	[98]
pKGB5	pKNT25 with modified *mcs*	[98]
pMEB3	pET28mod derivative, containing *PA2577* gene inserted as EcoRI-SacI fragment amplified with the use of #1 and #2 primers to obtain His_6_-*PA2577* fusion	This study
pMEB6	pAKE600 derivative with upstream region of *PA2577* gene inserted as EcoRI-HindIII fragment amplified with the use of #3 and #4 primers	This study
pMEB11	pBGS18 derivative with downstream region of *PA2577* gene inserted as HindIII-BamHI fragment amplified with the use of #5 and #6 primers	This study
pMEB12	pKGB8 derivative, containing *PA2577* gene under the control of *BAD*p promoter; *PA2577* gene from pMEB3 inserted as EcoRI-SacI fragment	This study
pMEB17	pAKE600 derivative with fused upstream and downstream region of *PA2577* gene; downstream region from pMEB11 inserted into pMEB6 as HindIII-BamHI creating up and down fusion linked with HindIII	This study
pMEB58	pKGB4 derivative containing *PA2577* gene without stop codon fused with T18; *PA2577* amplified with the use of primers #1 and #18 inserted as EcoRI-KpnI fragment	This study
pMEB61	pKGB5 derivative containing *PA2577* gene without stop codon fused with T25; *PA2577* amplified with the use of primers #1 and #18 inserted as EcoRI-KpnI fragment	This study
pMEB64	pAMB9.37 derivative with inserted EcoRI-XhoI fragment containing *PA2577* gene (cloned from pMEB3) under control of *tac*p promoter	This study
pMEB67	pLKB2 derivative encoding T25-*PA2577* translational fusion; *PA2577* gene from pMEB64 inserted as EcoRI-KpnI fragment	This study
pMEB69	pLKB4 derivative encoding T18-*PA2577* translational fusion; *PA2577* gene from pMEB64 inserted as EcoRI-KpnI fragment	This study
pMEB78	pCM132 derivative with 192 bps fragment containing intergenic region upstream of *PA2576* amplified with the use of #16 and #17 primers fused with *lacZ* to obtain transcriptional fusion	This study
pMEB79	pKAB240 derivative with *PA2577* gene fused with flag-tag; *PA2577* gene without stop codon amplified with the use of #1 and #7 primers inserted as EcoRI-XhoI fragment	This study
pMEB83	pAMB9.37 with modified *mcs* (EcoRI-PstI-SmaI-BglII-SalI inserted in the place of EcoRI-XhoI fragment)	This study
pMEB84	pMEB83 with inserted EcoRI-SmaI fragment from pMEB79 containing *PA2577*-flag fusion	This study
pMEB105	pET28mod derivative, containing *PA2577* gene inserted as NcoI-XhoI fragment amplified with the use of #8 and #9 primers to obtain *PA2577*-His_6_ fusion	This study
pMEB121	pKGB5 derivative containing *PA2576* gene without stop codon fused with T25; *PA2576* amplified with the use of primers #19 and #24 inserted as EcoRI-BamHI fragment	This study
pMEB122	pLKB2 derivative encoding T25-*PA2576* translational fusion; *PA2576* gene inserted as EcoRI-BamHI fragment	This study
pMEB156	pAKE600 derivative with upstream region of *PA2576* gene inserted as EcoRI-HindIII fragment amplified with the use of #10 and #11 primers	This study
pMEB157	pBGS18 derivative with downstream region of *PA2576* gene inserted as HindIII-BamHI fragment amplified with the use of #12 and #13 primers	This study
pMEB164	pAKE600 derivative with fused upstream and downstream region of *PA2576* gene; downstream region from pMEB157 inserted into pMEB156 as HindIII-BamHI creating up and down fusion linked with HindIII	This study
pMEB185	pAMB9.37 derivative with inserted EcoRI-KpnI fragment containing *PA2578* gene amplified with the use of #25 and #26 under control of *tac*p promoter	This study
pMEB186	pAMB9.37 derivative with inserted EcoRI-KpnI fragment containing *PA2577*–*PA2578* operon amplified with the use of #1 and #26 under control of *tac*p promoter	This study
pMEB189	pPTOI derivative with 192 bps fragment containing intergenic region upstream of *PA2576* amplified with the use of #16 and #17 primers fused with *xylE* to obtain transcriptional fusion	This study
pMEB201	pAMB9.37 derivative with inserted EcoRI-XhoI fragment containing *PA2576* gene amplified with the use of #19 and #22 primers under control of *tac*p promoter	This study
pMEB239	pLKB4 derivative encoding T18-*PA3694* translational fusion; *PA3694* gene inserted as EcoRI-SacI fragment	This study
pMEB240	pLKB4 derivative encoding T18-*PA5006* translational fusion; *PA5006* gene inserted as BamHI-SacI fragment	This study
pMEB242	pLKB2 derivative encoding T25-*PA5006* translational fusion; *PA5006* gene inserted as BamHI-SacI fragment	This study
pMEB246	pKGB4 derivative containing *PA5006* gene without stop codon fused with T18; *PA5006* amplified with the use of primers #29 and #31 inserted as BamHI-SacI fragment	This study
pMEB247	pKGB5 derivative containing *PA5006* gene without stop codon fused with T25; *PA5006* amplified with the use of primers #29 and #31 inserted as BamHI-SacI fragment	This study
pMEB248	pKGB5 derivative containing *PA3694* gene without stop codon fused with T25; *PA3694* amplified with the use of primers #27 and #32 inserted as EcoRI-SacI fragment	This study
pMEB249	pKGB4 derivative containing *PA3694* gene without stop codon fused with T18; *PA3694* amplified with the use of primers #27 and #32 inserted as EcoRI-SacI fragment	This study
pMEB250	pLKB2 derivative encoding T25-*PA3694* translational fusion; *PA3694* gene inserted as EcoRI-SacI fragment	This study

**Table A2:** List of primers used in this study.

Primers Used to Amplify DNA Fragments for Cloning		
No.	Name	Sequence 5′-3′
#1	2577ESF	GC GAATTC ATGACCGATGACATCGACC
#2	2577ESR	GC GAGCTC GAACCAGCGTTGCAGGATTG
#3	2577EHuF	GC GAATTC CCGAATACGCAGCGCCAGAAC
#4	2577EHuR	GC AAGCTT TCATCAATCGGTCATGTCGGCCTCG
#5	2577HBdF	GC AAGCTT TGAGCGAGGCACTCCGCATGC
#6	2577HBdR	GC GGATCC GCCGTTGCGGCGGTCGAACAG
#7	2577EX	CG CTCGAG GGTCATCGGGGGGAGCCG
#8	2577NXF	GC CCATGG ATGACCGATGACATCGACC
#9	2577NXR	CG CTCGAG ACCGGAACCGGATCCGGTCATCGGGGGGAGCCG
#10	2576EHuF	GC GAATTC CGTTCTTCCAGCTTGCGCAG
#11	2576EHuR	GCC AAGCTT TCA ATCGTT CAT GGGCGCTCTC
#12	2576HBdF	GCC AAGCTT TGA GGCGCGGGGGAAAGAAAAAG
#13	2576HBdR	GC GGATCC GTACTGTTCTTCGAAGACCAG
#14	p2577EBF	GC GAATTC GCATGC CCAGCGCGGCACAGGCG
#15	p2577EBR	GC GGATCC CATGTCGGCCTCGAAAG
#16	p2576F	GC GAATTC GCATGC GGGACAGTCGCGAGTCAGC
#17	p2576R	GC GGATCC GGCGCTCTCCCGCAAGACAG
#18	2577EK	CG GGTACC C GGTCATCGGGGGGAGCCG
#19	2576Ef	CG GAATTC ATGAACGATCCGATCCGTC
#20	BADNsiIF	ACGGATGGCCTTTATGCATTTCTACAAACT
#21	CM132RCy5	Cy5-CTTCCACAGTAGTTCACCACC
#22	2576EXr	CG CTCGAG GAAAAGCCCCGCCTCGTCG
#23	2576EBrS	CG GGATCC GAAAAGCCCCGCCTCGTCG
#24	2576EBr-S	CG GGATCC C GCCGCGACGCACCGCCGGC
#25	2578EKf	CG GAATTC ATGCCCACACCCGGCACGG
#26	2578EKR	GC GGTACC GCTCCGTTCCAACCTGTAG
#27	3694ESf	CG GAATTC ATGAGCGACCGTAACGTAC
#28	3694ESr	AT GAGCTC AGACGGGTGATGTCTCCCT
#29	5006Bf	GC GGATCC ATGAGACTGGCCGAACTGC
#30	5006BSr	GA GAGCTC GGTGGTCGTCGCTTGTCGT
#31	5006BSr-S	AT GAGCTC C AGCCCCGGCGGCGTCGCCGA
#32	3694ESr-S	AT GAGCTC CTTTCACTTCCTGCGGGACT
Primers used in RT-qPCR analysis		
#33	rpsLF	CTCGGCACTGCGTAAGGTAT
#34	rpsLR	TGTGCTCTTGCAGGTTGTGA
#35	nadBF	CTACCTGGACATCAGCCACA
#36	nadBR	GGTAATGTCGATGCCGAAGT
#37	PA2576qF	CCTGGGAATCGTCCATACCG
#38	PA2576qR	CGCGACCGGATAGATGAAGG
#39	PA1698qF	GGAAAGCCAGCAGAAGCTC
#40	PA1698qR	GGAGAAGCCCTCCAGGTAAG
#41	PA3037qF	GACGAAAGCCTCGACCTGT
#42	PA3037qR	GTCGAGGGTGACGAACAGAC
#43	PA0807qF	CGACAACCTCAACGACACC
#44	PA0807qR	GTAGTCGGGGAAGGTGAACA
