# Supplementary material for: The Lrp/AsnC-Type Regulator PA2577 Controls the EamA-like Transporter Gene PA2576 in Pseudomonas aeruginosa
Source: Int J Mol Sci. 2021 Dec 12;22(24):13340. doi: 10.3390/ijms222413340 (PMC8707732; doi:10.3390/ijms222413340)
Supplement: Supplementary file 1 [file ijms-22-13340-s001.zip › Supplementary figures PA2577 IJMS.pdf]

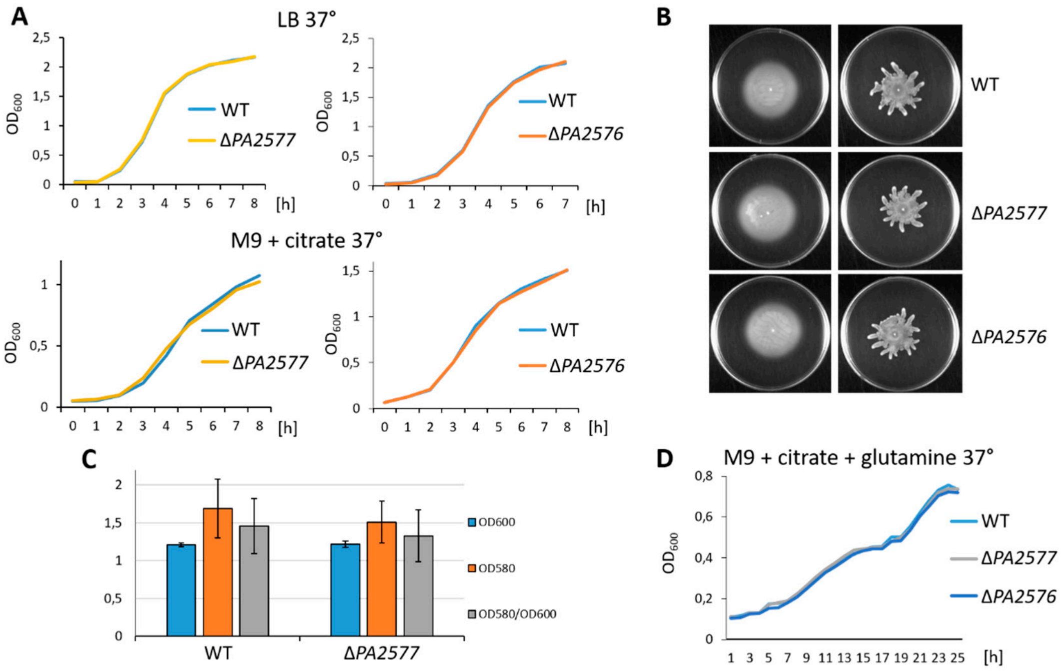

Figure S1. Phenotypic analysis of *P. aeruginosa* PAO1161 WT, Δ*PA2577* and Δ*PA2576* strains. Selected charts presenting growth curves, swimming and swarming assays and biofilm formation ability tests of *P. aeruginosa* PAO1161 WT, Δ*PA2577* and Δ*PA2576*. (**A**) Growth curves of the *P. aeruginosa* PAO1161 Δ*PA2577*, Δ*PA2576* and WT strains in LB or M9+citrate at 37°C (*leu*$^{+}$ strains). (**B**) Selected pictures of swimming (left panel) and swarming (right panel) assays performed for PAO1161 Δ*PA2577*, Δ*PA2576* and WT strains. (**C**) Biofilm production of PAO1161 WT and Δ*PA2577* in static cultures in LB medium. OD_{600} was measured and biofilm formation was assessed by crystal violet staining, followed by measurement of OD_{580}. Data represent mean OD_{600}, OD_{580} and OD_{580}/OD_{600} ratio ± SD from 5 biological replicates. (**D**) Growth curves of the PAO1161 Δ*PA2577*, Δ*PA2576* and WT strains in M9+citrate and glutamine at 37°C.

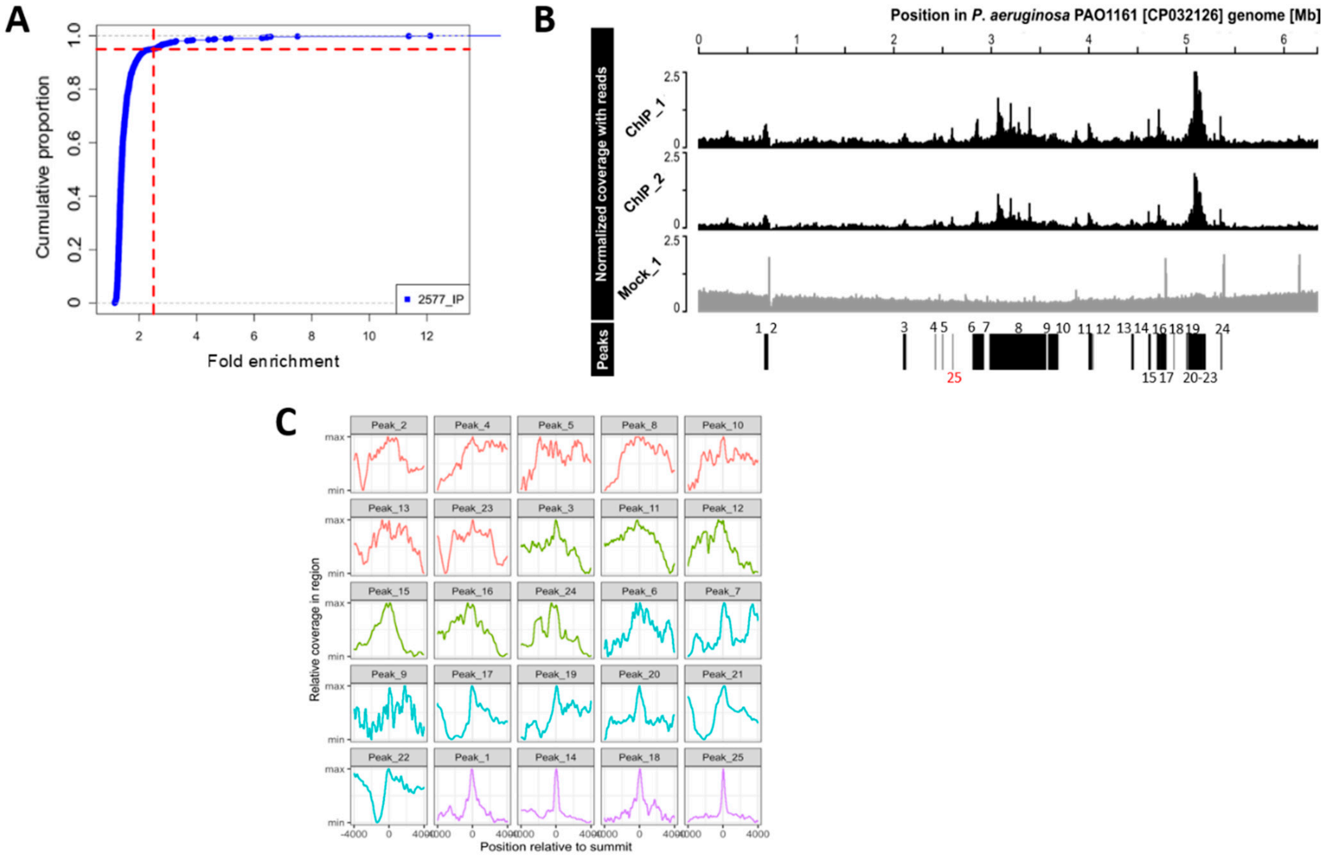

Figure S2. Identification of PA2577 binding sites in *P. aeruginosa* genome.
(**A**) Distribution of fold enrichment (FE) values for detected ChIP-seq peaks for cells expressing PA2577-FLAG. Cut-off value of 2.5 is shown as the red line. (**B**) Distribution of of PA2577 binding sites in PAO1161 genome. Data represent normalized coverage and location of identified peaks. (**C**) Analysis of the shape of identified ChIP-seq peaks. The normalized coverage signal was standardized to minimum and maximum value in the range +/- 4000 bp from predicted summit and the peaks were grouped by k-means clustering into 4 clusters (indicated with different color of lines). The details of peaks are included in Table S2.

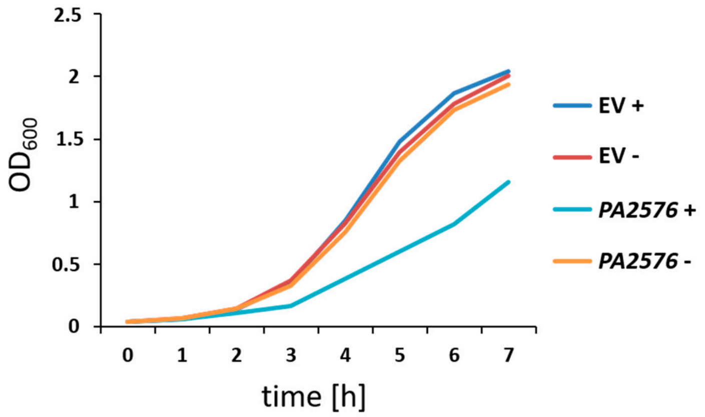

Figure S3. Impact of *PA2576* overexpression on the growth of the PAO1161 cells.
Growth curves of *P. aeruginosa* PAO1161 strains carrying pMEB201 (*lacI*^{Q}-*tac*p-*PA2576*; PA2576 overproducer) and pAMB9.37 (*lacI*^{Q}-*tac*p; EV control) grown in LB under selection with the addition of 0.5 mM (+) or absence (-) of IPTG.

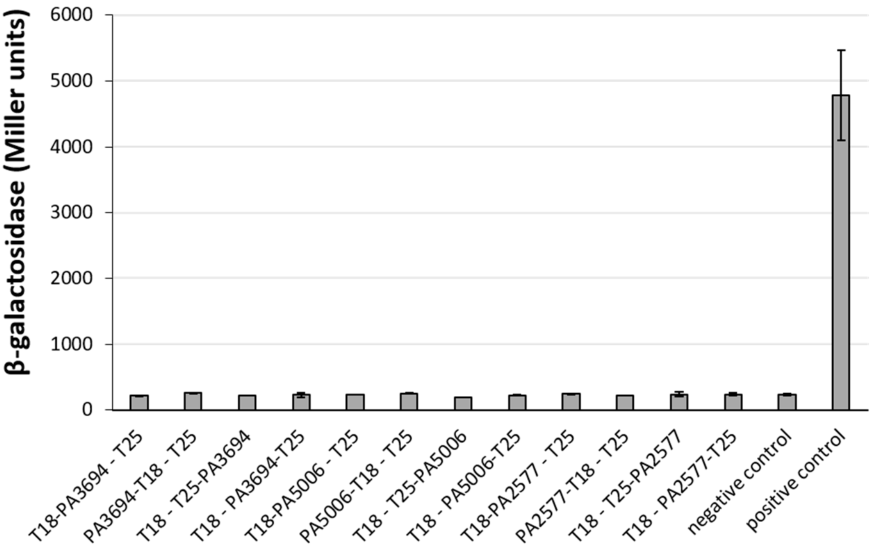

Figure S4. Control experiments for BACTH analysis. β-galactosidase measurements of *E. coli* BTH101 *cya*⁻ double transformants carrying empty BACTH vectors and appropriate proteins fused with *cyaA* subunit being the controls for the interactions identified by BACTH system. Negative control (empty pKT25 and pUT18C) and positive control (pKT25-zip and pUT18C-zip) (BACTH System Kit; Euromedex) are included.

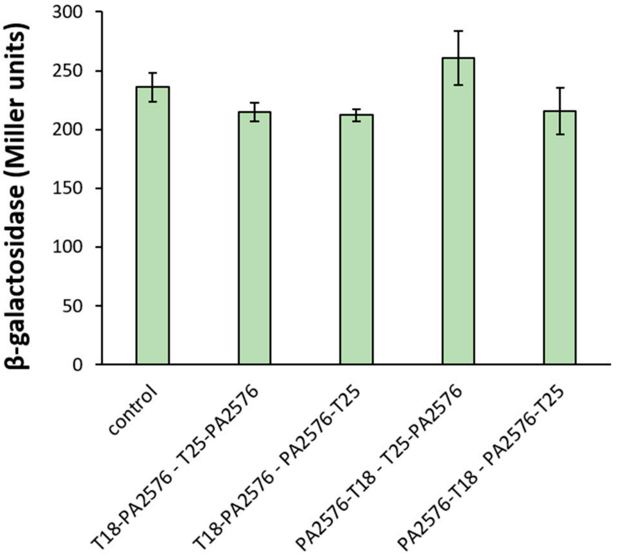

Figure S5. BACTH system analysis of PA2576 self-interactions. Data represent the mean ± SD values of β-galactosidase activity measured in cell extracts from three independent cultures of *E. coli* BTH101 *cya*⁻ double transformants producing indicated fusion proteins.
